# Pollutant Concentration Changes During the COVID-19 Lockdown in Barcelona and Surrounding Regions: Modification of Diurnal Cycles and Limited Role of Meteorological Conditions

**DOI:** 10.1007/s10546-021-00679-1

**Published:** 2021-12-27

**Authors:** Miguel García-Dalmau, Mireia Udina, Joan Bech, Yolanda Sola, Joan Montolio, Clara Jaén

**Affiliations:** 1 Departament de Física Aplicada–Meteorologia, Universitat de Barcelona, Barcelona, Spain; 2 DT Catalonia, AEMET, Barcelona, Spain; 3 Institute of Environmental Assessment and Water Research (IDAEACSIC), Barcelona, Spain

**Keywords:** Air quality, Boundary-layer height, COVID-19, Lockdown

## Abstract

One of the consequences of the COVID-19 lockdowns has been the modification of the air quality in many cities around the world. This study focuses on the variations in pollutant concentrations and how important meteorological conditions were for those variations in Barcelona and the surrounding area during the 2020 lockdown. Boundary-layer height, wind speed, and precipitation were compared between mid-March and April 2016–2019 (pre-lockdown) and the same period in 2020 (during lockdown). The results show the limited influence of meteorological factors on horizontal and vertical dispersion conditions. Compared with the pre-lockdown period, during lockdown the boundary-layer height slightly increased by between 5% and 9%, mean wind speed was very similar, and the fraction of days with rainfall increased only marginally, from 0.33 to 0.34, even though April 2020 was extremely wet in the study area. Variations in nitrogen dioxide ($\hbox {NO}_{{2}}$), particulate matter with a diameter less than 10 ${\mu }$m (PM10), and ozone ($\hbox {O}_{{3}}$) concentrations over a 10-year period showed a 66% reduction in $\hbox {NO}_{{2}}$, 37% reduction in PM10, and 27% increase in $\hbox {O}_{{3}}$ at a traffic station in Barcelona. The differences in the daily concentration cycle between weekends and weekdays were heavily smoothed for all pollutants considered. The afternoon $\hbox {NO}_{{2}}$ peak at the traffic station was suppressed compared with the average daily cycle. The analysis of ozone was extended to the regional scale, revealing lower concentrations at rural sites and higher ones in urban zones, especially in Barcelona and the surrounding area. The results presented not only complement previous air quality COVID-19 lockdown studies but also provide insights into the effects of road-traffic reduction.

## Introduction

The lockdown that took place in Spain from mid-March to April 2020 due to the COVID-19 outbreak caused many economical activities to stop and, as a consequence, the reduction of emissions of primary pollutants in the atmosphere, including nitrogen oxides ($\hbox {NO}_{{x}}$) and particulate matter with a diameter less than 10 ${\mu }$m (PM10). The immediate consequences were a general improvement in air quality (Habibi et al. 2020), although large parts of the population in urban areas who are at risk were still breathing air that does not meet the air quality guidelines of the World Health Organization (WHO).

Atmospheric pollutant concentrations are highly dependent on the amount of emissions, although meteorological factors, such as wind speed and direction, solar radiation, precipitation, and temperature inversions also influence air pollutant levels. More specifically, the capacity of the atmosphere to cause the mixing, dispersion, and dilution of pollutants in the air is mainly driven by the wind speed and the boundary-layer depth (e.g., Emeis and Schäfer 2006; Sicard et al. 2006; Lee et al. 2019; Xiang et al. 2019; Udina et al. 2020; Yang et al. 2020). If the wind speed increases, pollutant concentrations decrease because the pollutants are dispersed and diluted. Besides, for a given wind speed, the planetary boundary-layer depth, (PBL) $(z_i)$ determines the volume of air where pollutant species can be diluted, so that higher (lower) pollutant concentrations would be expected with shallower (deeper) PBLs. In order to estimate the PBL depth, several methods can be applied using atmospheric radiosondes and ceilometers (Eresmaa et al. 2006; Hennemuth and Lammert 2006; Seidel et al. 2010; Lotteraner and Piringer 2016; Kotthaus and Grimmond 2018; García-Franco et al. 2018) and the results have been used to explain the air pollutant concentrations (Pandolfi et al. 2013; Lee et al. 2018, 2019).

In addition to primary pollutants, $\hbox {O}_{{3}}$, a secondary pollutant formed in complex chemical reactions involving $\hbox {NO}_{{x}}$ and volatile organic compounds (VOCs) in the presence of high ultraviolet irradiance, can be strongly affected by a reduction in emissions. Tropospheric $\hbox {O}_{{3}}$ surface concentrations depend on the type of area (rural or urban) and on variations in precursors. Basically there are two ozone sensitivity regimes regulated by the ratio between $\hbox {NO}_{{x}}$ and VOCs, and ozone may react to changes in both VOC and $\hbox {NO}_{{x}}$ concentrations. In rural or suburban areas, where VOCs are dominant, ozone increases when $\hbox {NO}_{{x}}$ increases. On the other hand, in urban areas where $\hbox {NO}_{{x}}$ are dominant, ozone may increase if $\hbox {NO}_{{x}}$ are reduced (Sillman and He 2002).

Many authors have explored the consequences of COVID-19 lockdowns on air quality around the world, in relation to reducing the global burden of disease (Venter et al. 2020; Chauhan and Singh 2020; Nie et al. 2021). Most urban areas experienced a reduction in primary air pollutants during lockdown (Habibi et al. 2020; Burns et al. 2021; Velders et al. 2021; He et al. 2021). In China, $\hbox {NO}_{{2}}$ levels were reduced globally by 30% (He et al. 2020; Wang et al. 2021; Liu et al. 2020) and even 53% in northern regions (Shi and Brasseur 2020; Li et al. 2020; Chu et al. 2021). In the city of Wuhan, particulate matter was reduced (Sulaymon et al. 2021) by 53% according to Sicard et al. (2020). Shi and Brasseur (2020) showed an increase in ozone concentration by a factor of $2.2 \pm 0.2$ after a $\hbox {NO}_{{2}}$ reduction of 54 ± 7%. In Delhi, India, a reduction of 50% of PM10 and PM2.5 was reported (Mahato et al. 2020; Dumka et al. 2021). In Portugal, Gama et al. (2020) found a higher reduction in $\hbox {NO}_{{2}}$ (41%) than for PM10 (18%), which was more significant at traffic stations ($\hbox {NO}_{2}>$ 60%), while greater reductions in PM10 occurred in Poland, up to a maximum of 34% (Filonchyk et al. 2020). In Spain, early results after the lockdown including a 15-day analysis concluded that there was a 51% reduction in $\hbox {NO}_2$ and a 30% reduction in PM10 in Barcelona in comparison with levels recorded during the previous months (Tobías et al. 2020). Baldasano (2020) reported an average reduction in $\hbox {NO}_{{2}}$ of 62% in Madrid and 50% in Barcelona during March 2020. Petetin et al. (2020) calculated an average reduction of 50% in $\hbox {NO}_{{2}}$ during lockdown over Spain, using machine learning, while Mesas-Carrascosa et al. (2020) showed a significant correlation between population activity and the reduction in NO${_2}$ in Spain using TROPOspheric Monitoring Instrument (TROPOMI) of Sentinel-5P images. Briz-Redón et al. (2020) showed a greater reduction in $\hbox {NO}_{{2}}$ in the most heavily populated cities of Spain, a less significant reduction in PM10, and an increase in $\hbox {O}_{{3}}$.

Most previous studies based their analysis on the comparison of pollutant concentrations before and after lockdown in different locations around the world. Some of them (e.g., Shi and Brasseur 2020; Tobías et al. 2020) mentioned the need to include detailed meteorological information for a complete understanding of the observed variations. Recent studies included meteorological variables to assess their influence on the measured concentration levels using machine learning with data from ERA5 reanalysis (Petetin et al. 2020), adjusting for seasonality and meteorology temporally (Goldberg et al. 2020; Hörmann et al. 2021) or using a linear regression model with observations from surface stations (Briz-Redón et al. 2020). However, to the best of our knowledge, none have addressed the influence of meteorological factors including the horizontal and vertical dispersion conditions above the surface, and none have used several years for the comparison between average conditions before and during lockdown. Therefore, the aim of this paper is to determine the derived effects of the lockdown measures on air pollution concentrations considering the influence of meteorological factors on dispersion conditions from a local-scale perspective. The concentrations of $\hbox {NO}_{{2}}$, PM10, and $\hbox {O}_{{3}}$ were explored from mid-March to April 2020 in comparison with measurements from the previous 10 years in Barcelona and the surrounding area (north-east Iberian Peninsula). Meteorological factors included the wind speed at different heights, boundary-layer depth, and precipitation.

Section 2 describes the methodology employed, with a brief explanation of the study area, the datasets and the analytical methods for meteorological factors. Section 3 presents the results divided into two parts: (i) the analysis of meteorological conditions using parameters to establish the horizontal and vertical dispersion factors and (ii) the variation in air pollutant concentrations during the lockdown, including the daily variation between and within days and the effects on tropospheric ozone. The results are discussed in Sect. 4 and conclusions are presented in Sect. 5.

**Fig. 1:**
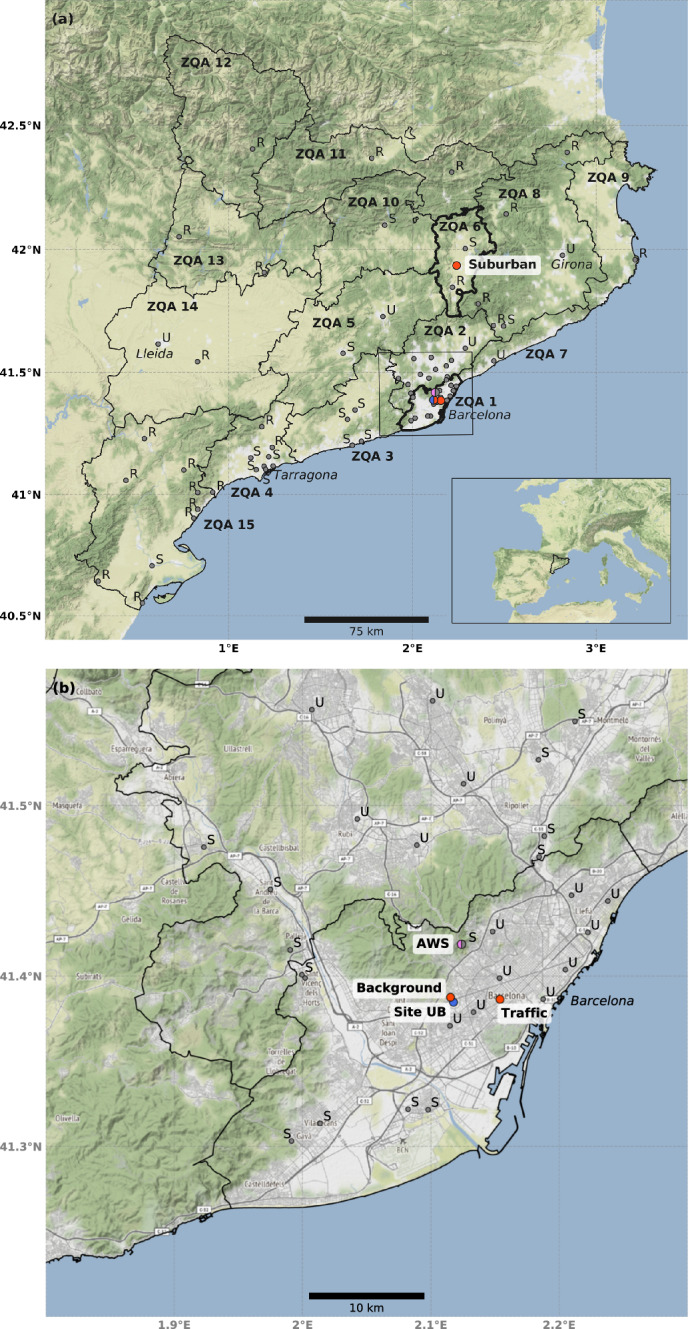
**a** Location of the area of study, 15 air quality zones (ZQA), type of measurement stations (U = urban, S = suburban, R = rural) and sites selected for the study (orange dots), including the suburban site Vic; **b** Enlargement of ZQA 1 with urban traffic and urban background stations (orange dots), UB site (blue dot) corresponding to the launch site of the radiosonde and the ceilometer location. The Automatic Weather Station (AWS) (pink dot) corresponds to the automatic meteorological station at the Observatori Fabra

## Material and Methods

### Study Area

Figure 1 shows the area of study, centred in Catalonia in the north-east of the Iberian Peninsula. The regional administration manages a dense air quality network (XVPCA) with 78 automatic stations and 106 manual stations. The territory is divided into 14 air quality zones (ZQA) with similar emission and dispersion conditions, within which there is at least one pollutant measurement station per zone. The stations are classified according to the degree of urbanization —urban (U), suburban (S) or rural (R)— and the dominant pollution source —background (B), industrial (I) or traffic (T) (XVPCA 2020). They measure the most critical pollutants in Catalonia ($\hbox {NO}_{{2}}$, PM10, and tropospheric $\hbox {O}_{{3}}$, among others). In the region of study there are two areas where the legal thresholds of pollutants are usually exceeded: i) the Barcelona urban area and its surroundings ($\hbox {NO}_{{2}}$ and PM10), and ii) the Vic plane and its surroundings, where $\hbox {O}_{{3}}$ peaks occur during summer when the sea breeze transports ozone precursors from urban regions inland to the rural plane (Jaén et al. 2021). Therefore, the XVPCA measurement stations used to analyze the air quality in this study included two urban stations in Barcelona: a traffic station (Eixample) and a background station (Palau Reial), hereafter called traffic and background respectively, and a suburban inland station in Vic (hereafter called suburban) —see Fig. 1 and Table 1.

**Table 1:** Air quality network automatic measurement stations (XVPCA)

Class	Area, Type	Location	Location	Pollutants
Traffic	Urban, Traffic	Barcelona (Eixample)	41.385$^{\circ }$N, 2.154$^{\circ }$E	$\hbox {NO}_{\text {X}}$, PM10, $\hbox {O}_{{3}}$
Background	Urban, Background	Barcelona (Palau Reial)	41.387$^{\circ }$N, 2.116$^{\circ }$E	$\hbox {NO}_{\text {X}}$, PM10, $\hbox {O}_{{3}}$
Suburban	Suburban, Background	Vic (Estadi)	41.934$^{\circ }$N, 2.240$^{\circ }$E	PM10, $\hbox {O}_{{3}}$

### Data

The meteorological study was performed using data from atmospheric radiosondes, a ceilometer, and an automatic surface meteorological station. The air quality was analyzed using the XVPCA network data. The comparison involving meteorological variables was based on two periods: i) from 14 March to 30 April of 2016–2019 (combining 4 years of data), called the “pre-lockdown" (hereafter PLD) period and ii) from 14 March to 30 April 2020, called the “during lockdown" (hereafter DLD) period. The comparison of pollutant concentrations used data from the same months from the PLD period over 10 years, from 2010 to 2019, while the DLD period was the same.

Atmospheric radiosondes (RSD) located at the UB site (see Fig.1b) provided the vertical atmospheric profile. Data of RSD were obtained from the Barcelona radiosonde station, operated by the Meteorological Service of Catalonia and located in the Faculty of Physics of the University of Barcelona (41.385$^{\circ }$N, 2.118$^{\circ }$E) at 98 m above sea level (a.s.l.). The Barcelona RSD has a long time series, beginning in 1998 and has been integrated into the Global Meteorological Network since 2008. It has been running automatically since 2013 with the Meteomodem M10 radiosonde model (Meteomodem 2021). In this study, we use the midday RSD data from 14 March to 30 April 2016 to 2020, considering 1100 UTC as the launching time. The temperature and specific humidity profiles were used to estimate the PBL depth (see Sect. 2.3). The wind speed was used to provide information about horizontal dispersion in the vertical atmospheric layers within the boundary-layer.

A laser ceilometer (CL-31, Vaisala Inc., Finland) has been in operation at the UB site since August 2015. It is equipped with an InGaAs diode laser that sends 910 nm light pulses. It has a measurement range up to 7.6 km, 10-m vertical resolution and a temporal resolution of 16 s, providing vertical backscattering profiles. The PBL depth was computed using the Vaisala Boundary-Layer View software (BL-VIEW), which estimates up to three planetary boundary layers and cloud heights using the enhanced gradient method averaging every 10 min, automatically filtering out rainy conditions (VAISALA 2020). The PBL depth estimated from ceilometer data is used from mid-March to April (2016–2020) at 1110 UTC and is compared with the estimations from the RSD data, as the estimated nearest time when the sonde of the RSD reached the PBL top.

In order to ensure the PBL depth was only computed in non-rainy conditions, precipitation data were obtained from the station also located on the UB site. The wind speed (*VV*) and rainfall data (*RAIN*) from an automatic surface weather station (AWS) located at Barcelona Observatori Fabra (41.419$^{\circ }$N, 2.124$^{\circ }$E) were also used. The station is located on the north-western edge of Barcelona, on a mountain slope about 400 m a.s.l., and therefore is less influenced by the urban environment. Meteorological analysis including PBL depth, *VV*, and *RAIN* was performed for the two considered temporal periods, PLD (2016–2019) and DLD (2020).

### Applied Methods

In order to estimate vertical dispersion conditions, the PBL depth was computed for days without rain for values before and during lockdown. The PBL top establishes the upper limit of the air volume in which the pollutants accumulate. During the daytime, the mixed layer is characterized by profiles of constant potential temperature, humidity, and refractivity. The PBL depth was calculated using two different algorithms.

The first method used (hereafter RAOB) is based on the methodology of Wang and Wang (2014) called the “Gradient Detector" methodology, including modifications by Yuval et al. (2020). This algorithm uses RSD data, computing the gradients of potential temperature ($\theta$), specific humidity (*q*), relative humidity (*RH*), and refractivity (*N*) with a fourth-order discrete-derivative approximation. The specific humidity is calculated as

1 $$\begin{aligned} q=\frac{r_{d}}{r_{v}}\frac{e}{p-e}, \end{aligned}$$

where $r_{d}=$ 287.05 J kg$^{-1}$ K$^{-1}$ and $r_{v}=$ 461.50 J kg$^{-1}$ K$^{-1}$ are the specific gas constants for dry air and water vapour respectively, *e* is the water vapour pressure, calculated using $e=RH e_s$, $e_s$ is the saturation water vapour pressure, computed from the Magnus–Tetens expression, and *p* is the atmospheric pressure. Refractivity was computed as (Bech et al. 2003)

2 $$\begin{aligned} N=77.6\frac{p}{T}+373000\frac{e}{T^{2}}, \end{aligned}$$

where *p* is the pressure (in hPa) and *T* is the air temperature (in K). As the typical temperature and humidity profiles show sharp variations at the top of the boundary layer, the PBL top is identified as the height at which the maximum gradient of $\theta$ and minima gradients of *q*, *RH* and *N* are located. Gradient peaks were located using an automated function from Scipy software (Virtanen et al. 2020), equipped with a quality filter to dismiss unreliable PBL depths. To provide high quality values, PBL depths lower than 250 m were discarded.

The second method used was the BL-VIEW Enhanced Gradient method (VAISALA 2020) followed by a selection algorithm, hereafter called CEIL. The Vaisala BL-View software estimates the boundary-layer height from the analysis of the vertical backscattering profile. More specifically, it is based on the so-called “Gradient Method”, which relates the highest negative gradient of the backscatter coefficient with the top of the PBL. The algorithm is enhanced with vertical and temporal averaging processes to reduce false-positive PBL depth identifications induced by clouds, precipitation and fog (Münkel and Roininen 2010). The result is a robust algorithm that detects up to three possible PBL depth estimates from the same vertical profile. We used this criterion and then selected the candidate of PBL depth following the methodology of Lotteraner and Piringer (2016). The final PBL depth value was only considered when the meteorological station set on the UB site did not register precipitation during the previous 5 h (between 0600 UTC and 1110 UTC).

Horizontal dispersion was analyzed using data of wind speed from two sources. The wind speed from the AWS was used as a background reference (at 411 m a.s.l.). The wind speed obtained from RSD data was averaged for four layers from the surface up to the PBL top selected using the RAOB method according to the following height intervals

$$\begin{aligned} VV_{1}&= \overline{VV}(z) | z \in [0,0.1z_i), \\ VV_{2}&= \overline{VV}(z) | z \in [0.1z_i,0.25z_i),\\ VV_{3}&= \overline{VV}(z) | z \in [0.25z_i,0.5z_i),\\ VV_{4}&= \overline{VV}(z) | z \in [0.5z_i,z_i). \end{aligned}$$

In order to quantify pollutant removal from the atmosphere through wet deposition, precipitation data were gathered from the selected AWS.

The results are shown using boxplots, which present several statistics. The box size represents the inter-quartile distance from the 25th percentile (*Q*1) up to the 75th percentile (*Q*3), representing the inter-quartile range (*IQR*), which depends on the values and the dispersion of the plotted series. The median value of the points within the bin is shown using a horizontal line inside the box. The upper (lower) whisker extends to the maximum (minimum) datum position at $Q3 + 1.5IQR$ ($Q3 - 1.5IQR$). Outliers are marked with dots above/below the whiskers. In the wind speed boxplots, outliers are not shown to make the plots clearer.

## Results

The results are divided into two sections: the meteorological conditions and the pollutant concentration variation.

### Meteorological Conditions

**Fig. 2:**
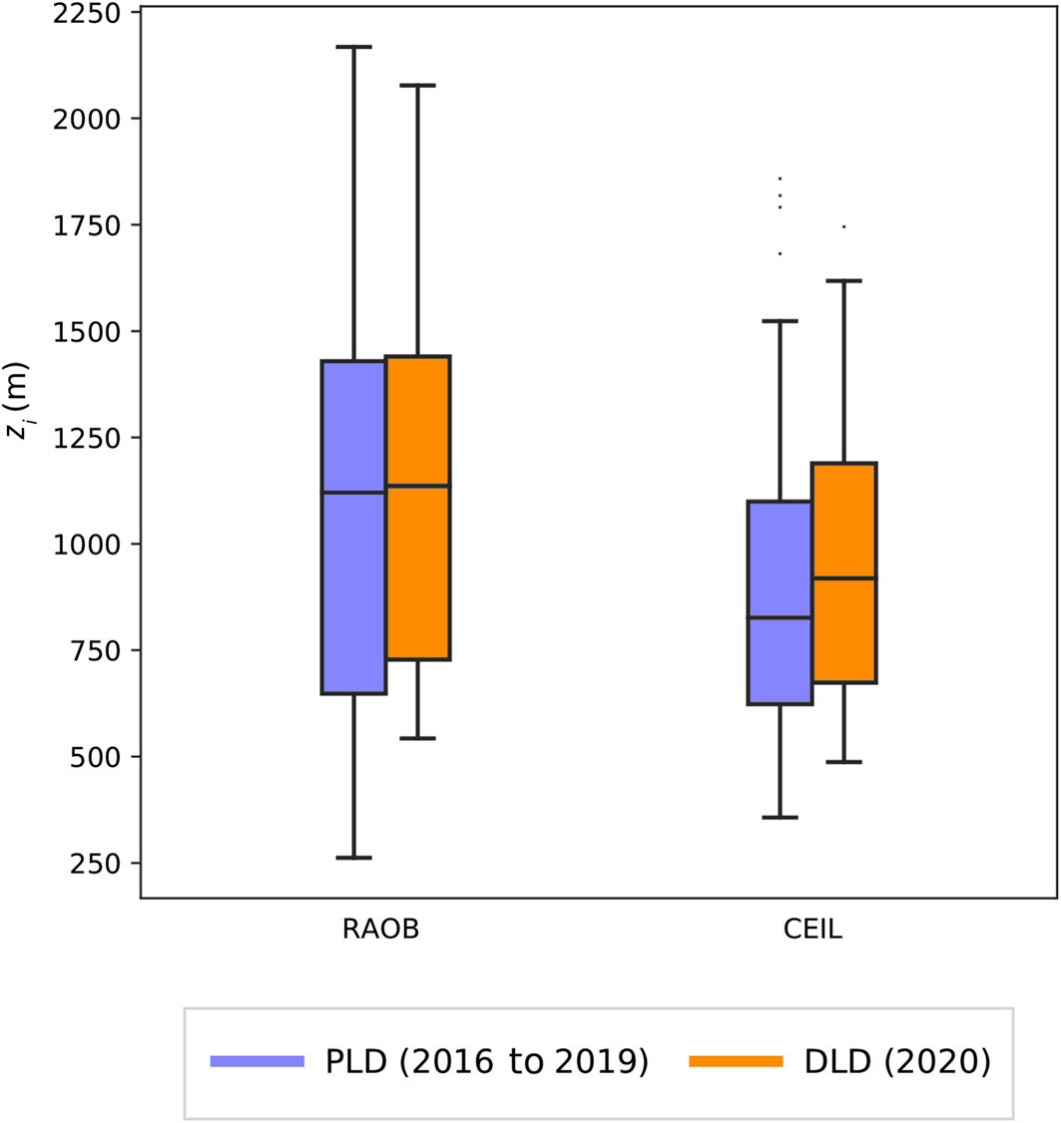
Boxplots of PBL depth $(z_i)$ computed for pre-lockdown (PLD) period (orange) and during lockdown (DLD) period (blue) using the RAOB and CEIL algorithms

Statistical data from the PBL depth obtained using the two algorithms, RAOB and CEIL, are summarized in Table 2 and Fig. 2. All days without rain were considered, including clear sky and cloudy days. Firstly, a comparison between the RAOB and CEIL methods shows that the PBL depth determined using the RAOB method were greater than those determined using the CEIL method. This is what we expected based on the characteristics of these algorithms, since RAOB methodology is very sensitive to humidity, temperature, and refractivity shifts, which is more likely to occur in higher layers. Moreover, the CEIL method tends to select lower PBL depth candidates since the quality of backscattering signal decreases with height. Regarding the difference between the pre-lockdown (PLD, 2016–2019) and during lockdown periods (DLD, 2020), the results for the RAOB algorithm showed a mean PBL depth of 1090 m and a standard deviation of 470 m for the PLD period, while the mean PBL depth was 1140 m, with a standard deviation of 450 m for the DLD period. There was, therefore, a slight increase in the PBL depth during lockdown of around 5%. Regarding the CEIL algorithm, a mean of 880 m with a standard deviation of 320 m was found during PLD period (2016–2019), and a mean PBL depth of 960 m and standard deviation of 340 m was found in the DLD period, representing an increase of 9% during lockdown weeks.

**Fig. 3:**
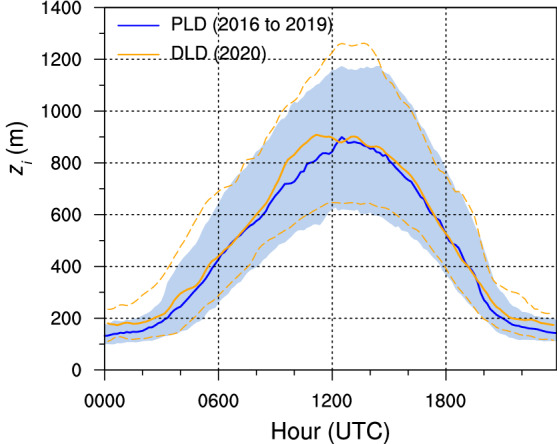
Daily cycle of the PBL depth $(z_i)$ from 14 March to 30 April for the 4 year PLD period (blue) and for 2020 in the lockdown (DLD) period (orange). Solid thick lines correspond to median PBLHs and 25th and 75th PBLH percentiles are delimited by the blue semi-transparent shaded area for the PLD period and by the dashed orange lines for the DLD period

In order to explore the PBL depth at times other than midday, we computed the daily cycle of the PBL depth obtained by the CEIL method (Fig. 3). In general, the median DLD PBL depth was close to the median PLD PBL depth, although some differences can be seen. At night, between 0000 and 0200 UTC, the PBL depth during the lockdown was greater than in the PLD period, reaching its 75th percentile, although the interquartile difference was very small. Another interesting deviation was observed around 0900 to 1200 UTC, when greater PBL depths were estimated for the DLD period. In both cases a deeper PBL would lead to more dilution of the pollutant species, which would have favoured the decrease in pollutant concentrations recorded during the lockdown period.

**Table 2:** Statistics (mean, standard deviation, and percentiles) of the PBL depth PBLH computed with the two algorithms, RAOB and CEIL, for March and April, comparing the PLD period 2016–2019 and the DLD period in 2020

Measurement	Mean (m)	Std (m)	5th (m)	25th (m)	50th (m)	75th (m)	95th (m)
PBLH RAOB PLD	1090	470	390	650	1120	1430	1860
PBLH RAOB DLD	1140	450	570	730	1140	1440	1980
PBLH CEIL PLD	880	320	450	620	830	1100	1430
PBLH CEIL DLD	960	340	490	670	920	1190	1570

**Fig. 4:**
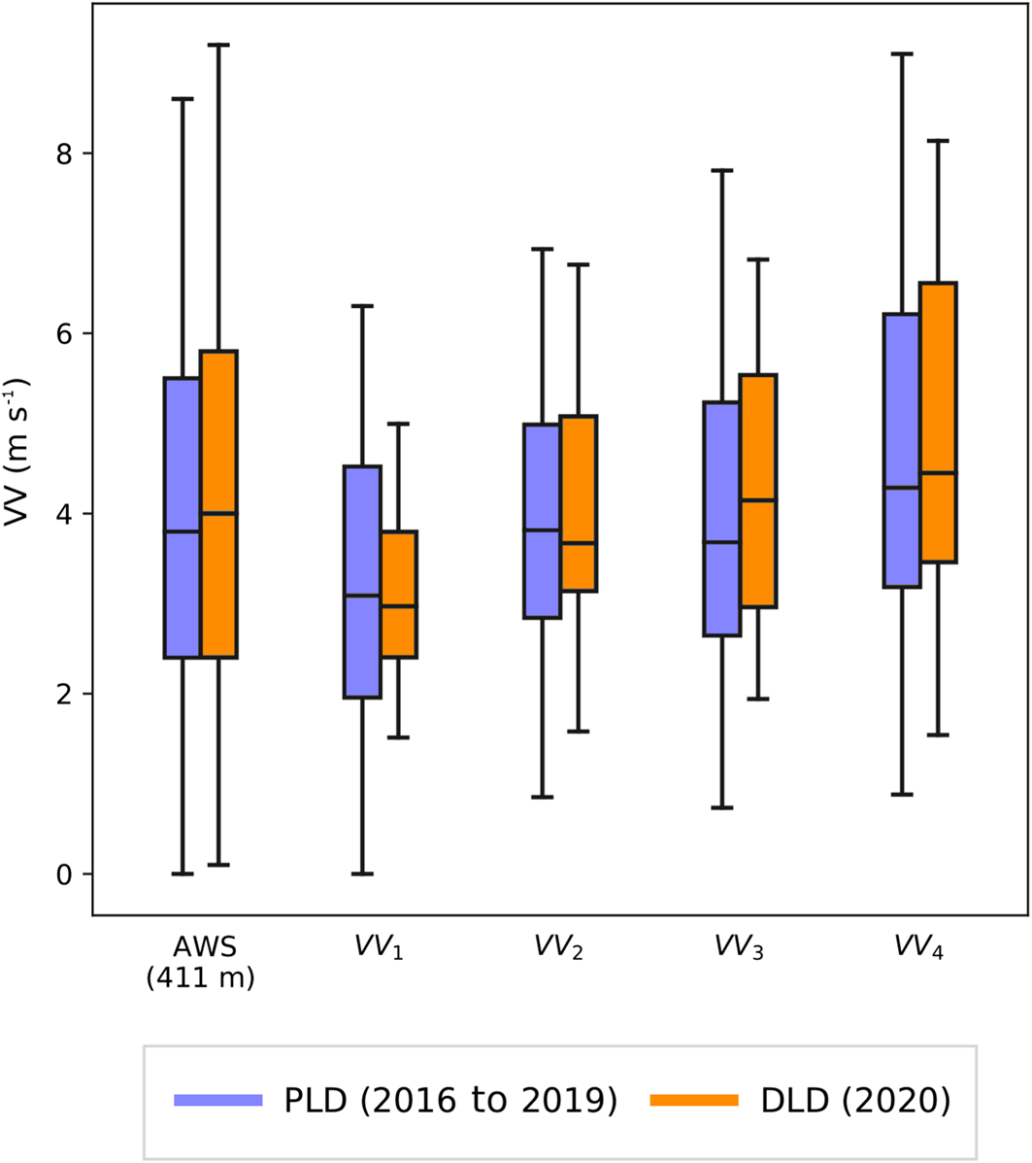
Boxplot of wind speed (*VV*) at the AWS station and at different layers within the boundary-layer ($VV_{i}$). Orange boxes represent data from the PLD period and blue boxes represent data from the DLD period

Regarding the wind speed (*VV*), similar statistics were obtained for both periods. The mean wind speed at the AWS station was almost the same in the DLD period, 4.3 m s$^{-1}$, as in the PLD period, 4.2 m s$^{-1}$, with similar standard deviations of 2.3 m s$^{-1}$ and 2.4 m s$^{-1}$ respectively. Analysis of different vertical layers (Fig. 4) reveals lower median VV values in the first two layers, from the surface up to the 25% percentile of the PBL depth during the DLD period than in the PLD period. In contrast, at heights between 25–50% of the PBL depth the wind speed increased in the DLD period. Therefore, dispersion condition near the surface were worse during lockdown and slightly better in the middle of the PBL. The 25th percentiles present modest differences, with higher values in the DLD period, while at high percentiles the pattern changed, and stronger winds occurred in the PLD, although this might partly reflect the larger amount of data analyzed in this case.

Rainfall also showed a similar pattern in the two time periods (Table 3). Rain was detected at the station roughly once every three days. However, during 2020, the amount of precipitation per rain episode was substantially higher: 17.4 mm day$^{-1}$ compared with 7.5 mm day$^{-1}$ in the 2016–2019 period. Additionally, the fraction of 30-min periods of rain (rainy 30-min/total 30-min) doubled in the DLD period (third column in Table 3). Overall, the data indicate longer and more abundant rain episodes in the 2020 period. In fact, April 2020 rainfall accumulation was the highest in 107 years of data (SMC 2020). However, the number of rainy days was the same in the PLD and DLD periods, leading to the likelihood of similar pollutant removal from the atmosphere.

**Table 3:** Rainfall statistics at the AWS meteorological station. A day is considered as rainy if more than 0.1 mm is measured

Period	Rainy day ratio	Half-hour rainy ratio	Total rainfall	Rainfall/Day	Rainfall/Rain day
	(Rainy days/total days)	(Rainy 30-min/total 30-min)	(mm)	(mm day$^{-1}$)	(mm day$^{-1}$)
PLD (2016–2019)	0.33 (63/192)	0.05 (485/9216)	118	2.5	7.5
DLD (2020)	0.34 (18/48)	0.11 (263/2305)	313	6.5	17.4

Total rainfall considers rainfall recorded from 14 March to 30 April in both PLD (471 mm divided by 4 years) and DLD periods

### Variations in Air Pollutant Concentrations During Lockdown

The differences in concentration of $\hbox {NO}_2$, PM10, and $\hbox {O}_3$ between the DLD period and the 2010–2019 PLD period were explored by analyzing the daily variation and the daily cycles of pollutant concentrations. Ozone variations were also analyzed from a regional point of view using spatial distribution maps.

#### Daily Variation in Barcelona

**Fig. 5:**
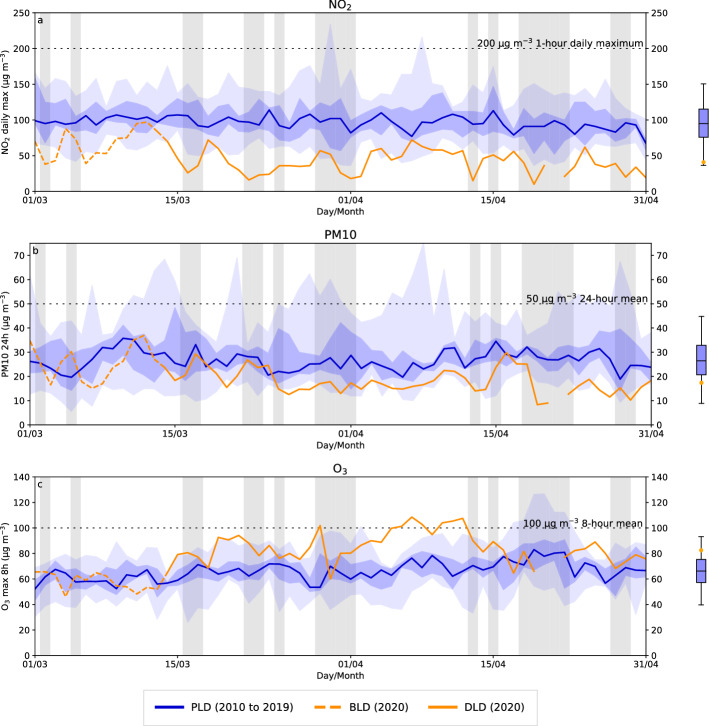
Pollutant concentrations: **a** $\hbox {NO}_{{2}}$ 1-h daily maximum, **b** PM10 daily mean, and **c** $\hbox {O}_{{3}}$ maximum of the 8-h rolling averages during March and April at the traffic station. Data shown correspond to the median of PLD period 2010–2019 (thick blue line) with the 25th and 75th percentiles (shaded dark blue) and the 2010–2019 daily minimum and maximum (shaded light blue), concentration during lockdown in 2020 (DLD) (solid orange line), and the first 13 days of March 2020 just before the lockdown in 2020 (BLD) (dashed orange line). Grey vertical areas identify rainy days during 2020. Horizontal lines represent WHO air pollution guidelines (until September 2021). Boxplots represent PLD period data (blue box and whiskers) including the DLD mean value (orange dot) on the same reference axis the corresponding main plot

$\hbox {NO}_{{2}}$ concentrations were strongly reduced in the DLD period at the traffic site (Fig. 5a). The daily mean concentrations recorded during March and April (57 $\upmu$ g m$^{-3}$) in the PLD period declined to 19 $\upmu$ g m$^{-3}$ in the DLD period, equivalent to a reduction of 66% (Table 4). From 14 to 16 March 2020, when the lockdown started, there was a sudden reduction in maximum 1-h $\hbox {NO}_{{2}}$ concentrations throughout the DLD period, in many cases far below the minimum values recorded in the PLD period. Throughout the DLD period the values remained below the WHO-recommended maximum concentration (200 $\upmu$ g m$^{-3}$). It can also be seen that the mean daily $\hbox {NO}_{{2}}$ maximum was located above the lower whisker of the PLD boxplot (Fig. 5a). In addition, rain occurred during the last week of March 2020, leading to a decrease in $\hbox {NO}_{{2}}$ concentrations, but there was no decrease in other rainy periods (see gray vertical areas in Fig. 5). The urban background monitoring station shows smaller absolute differences than the traffic site except for the $\hbox {NO}_{{2}}$ mean daily maximum (Table 4): $\hbox {NO}_{{2}}$ median values during the PLD period were around 30–40 $\upmu$ g m$^{-3}$ and DLD values were between 10 and 20 $\upmu$ g m$^{-3}$. The vast majority of DLD data was lower than the 25th percentile PLD. Nevertheless, the fractional reduction between the two time periods was similar to that at the traffic site, i.e., a 62% reduction in $\hbox {NO}_{{2}}$ levels (from 34 $\upmu$ g m$^{-3}$ to 13 $\upmu$ g m$^{-3}$).

PM10 concentrations were below the 25th percentile at both the traffic and background stations, except during the first lockdown week (Fig. 5b). The difference between the PLD and DLD periods was not as significant as it was for $\hbox {NO}_{{2}}$ but was gradual until the third week of March, when concentrations showed small variations between 15 $\upmu$ g m$^{-3}$ and 20 $\upmu$ g m$^{-3}$. After the third week of March, concentration were detected between the 75th percentile and the corresponding minimum of the 2010–2019 period except in the rainy third week of April, when the 2020 values marked a new minimum. The average PM10 concentration during the DLD period was lower than the 25th percentile of the PLD years, as seen in the boxplot in Fig. 5b.

In contrast to $\hbox {NO}_{{2}}$ and PM10 concentrations, ozone levels increase in the DLD period in Barcelona, especially at the traffic station (Fig. 5c). The daily maximum of 8-h rolling average of ozone increased to values above the PLD 75th percentile after 14th March, and stayed at this level for almost the entire lockdown period at the traffic station. In many cases the measured concentrations exceed the corresponding daily maximum 8-h rolling average of the 2010–2019 period. The last week of March and third week of April showed a local drop in ozone concentrations corresponding to rainy periods, which hinder ozone formation and lead to ozone removal from the atmosphere. The daily maximum of the 8-h rolling mean increased from 67 $\upmu$g m$^{-3}$ in the PLD period to 83 $\upmu$g m$^{-3}$ in the DLD period, a 23% increase, situating the DLD data above the 75th PLD percentile. In addition, during a short period in the April DLD period the WHO standard for the daily maximum of the 8-h rolling mean of ozone (100 $\upmu$g m$^{-3}$) was exceeded. A smaller increase was observed at the background station (not shown), where the increase compared to the PLD mean was 11%. Unlike at the traffic station, where the vast majority of values were higher than the 75th percentile, here they are found between the 50th percentile and the maximum during the PLD period. The observed ozone increase can be attributed to the already known sensitivity of the ozone regime in urban areas, where the VOCs/$\hbox {NO}_x$ ratio is relatively low, which leads ozone to increase when $\hbox {NO}_2$ decreases (Sillman and He 2002). This ozone regime sensitivity has been observed in other cities around the world (Shi and Brasseur 2020; Cazorla et al. 2021).

**Table 4:** Concentrations and relative differences (Rdiff) in % between the PLD (2010–2019) and DLD (2020) periods for the traffic and background stations, showing the mean of the daily mean concentration of $\hbox {NO}_{{2}}$ (first row), the mean of the 1-h daily maximum $\hbox {NO}_{{2}}$ (second row), the mean of the daily mean concentration of PM10 (third row), and the mean of the daily maximum of the 8-h rolling averages of $\hbox {O}_{{3}}$ (last row)

	Traffic			Background		
	PLD ($\upmu$g m$^{-3}$)	DLD ($\upmu$g m$^{-3}$)	RDiff (%)	PLD ($\upmu$g m$^{-3}$)	DLD ($\upmu$g m$^{-3}$)	RDiff (%)
$\hbox {NO}_{{2}}$ daily mean	57	19	$-$66	34	13	$-$62
$\hbox {NO}_{{2}}$ daily max	98	42	$-$57	79	32	$-$60
PM10 daily mean	28	18	$-$37	20	14	$-$31
$\hbox {O}_{{3}}$ 8-h max	67	83	+23	85	94	+11

**Fig. 6:**
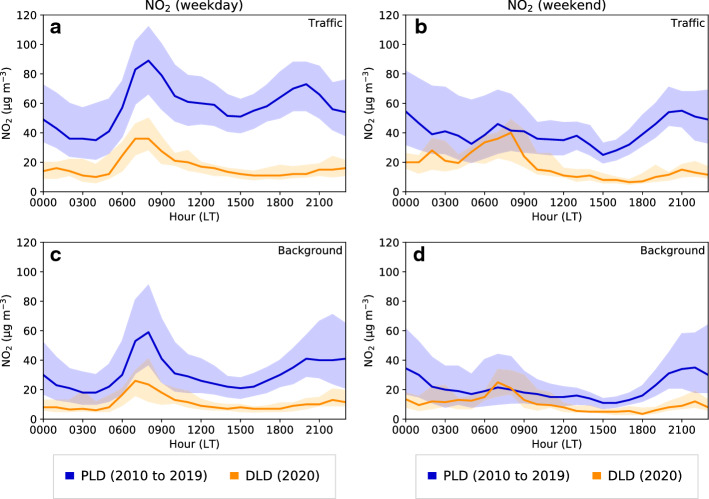
Daily $\hbox {NO}_2$ concentration cycle at traffic and background stations. Thick blue line corresponds to the median of the PLD period including data from mid March and April of 2010–2019 and the shaded blue limits mark the 25th and 75th percentiles. The thick orange line corresponds to the DLD median including data from mid March and April of 2020, and the shaded orange limits mark the 25th and 75th percentiles. Left panels correspond to weekdays and right panels to weekends. Local time (LT) corresponds to UTC $+$ 1)

#### Daily Cycle Variation

Figure 6 shows the averaged daily cycle of $\hbox {NO}_{{2}}$ concentrations at selected traffic and background locations on weekdays (left panels Fig. 6) and at weekends (right panels Fig. 6). A strong decline in $\hbox {NO}_{{2}}$ concentrations was observed during the 2020 lockdown in comparison with the 10-year mean. The morning peak around 0800 local time (LT, local time = UTC $+$ 1 h) was strongly reduced while the evening peak around 2000 LT was suppressed in the DLD period, mostly at the traffic station and on weekdays. Remarkable differences were also found in the maximum hourly value (morning/evening peak), as shown in Table 5. The traffic site showed a 58% decrease in the maximum of the hourly mean, from 90 $\upmu$g m$^{-3}$ to 37 $\upmu$g m$^{-3}$, whereas the background site showed a 63% reduction from 65 $\upmu$g m$^{-3}$ to 24 $\upmu$g m$^{-3}$.

**Table 5:** Mean and maximum of the mean concentration of $\hbox {NO}_{{2}}$, PM10, and $\hbox {O}_{{3}}$ in the PLD (2010–2019) and DLD (2020) periods, for weekdays (WD) and weekends (WK)

		Mean						Maximum of the mean					
		Traffic		Background		Suburban		Traffic		Background		Suburban	
		PLD	DLD	PLD	DLD	PLD	DLD	PLD	DLD	PLD	DLD	PLD	DLD
WD	$\hbox {NO}_{{2}}$ ($\upmu$g m$^{-3}$)	61	19	37	13			90	37	65	24		
	PM10 ($\upmu$g m$^{-3}$)	29	18	21	14			40	22	31	18		
	$\hbox {O}_{{3}}$ ($\upmu$g m$^{-3}$)	44	63	61	75	58	57	66	81	85	92	97	83
WK	$\hbox {NO}_{{2}}$ ($\upmu$g m$^{-3}$)	46	19	27	12			61	34	44	25		
	PM10 ($\upmu$g m$^{-3}$)	25	18	18	15			29	21	21	18		
	$\hbox {O}_{{3}}$ ($\upmu$g m$^{-3}$)	55	62	68	74	64	54	82	90	93	99	100	87

**Fig. 7:**
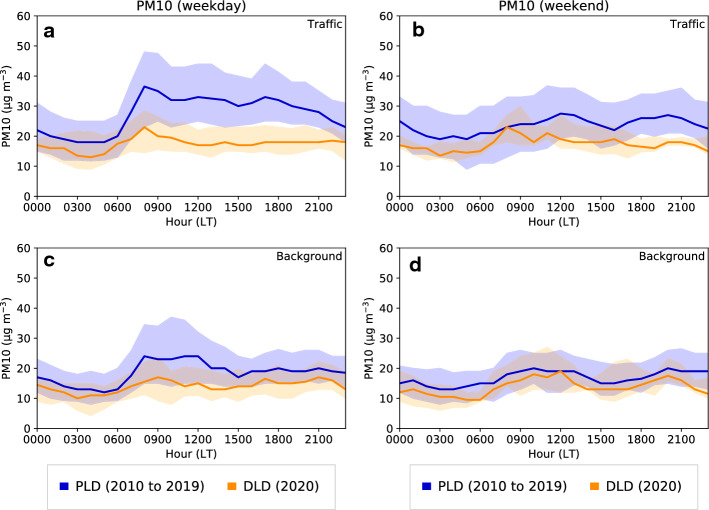
The same as in Fig. 6 but for PM10 concentrations

The daily cycles of PM10 for weekdays and weekends are shown in Fig. 7, for the traffic and background sites. The mean PM10 concentrations at the traffic station were reduced by 40% on weekdays during lockdown while the maximum morning mean was reduced by 49% (from 40 $\upmu$g m$^{-3}$ to 20 $\upmu$g m$^{-3}$). The differences were most remarkable from 0700 LT (LT, local time = UTC $+$ 1 h) to 2300 LT on weekdays, when the DLD median values were far below the 25th percentile of the PLD concentrations. At the background station, variations between periods were smaller, with a 35% reduction of the mean period value and a 44% reduction of the daily maximum.

In order to assess the possible influence of increased precipitation during the lockdown period, variations in pollutant concentration were calculated without considering hours of precipitation. The results show small variations in the mean concentrations presented in Table 5. On weekdays, the mean of the $\hbox {NO}_{{2}}$ daily maximum increased by only 1 $\upmu$g m$^{-3}$ at the traffic site, while PM10 stayed at the same mean concentrations. The largest change seen without considering the precipitation hours was in the maximum of the daily maximum of the 8-h rolling averages of $\hbox {O}_{{3}}$ at the background site at weekends, with an increase of 3 $\upmu$g m$^{-3}$. This indicates a residual effect of the pollutant wet deposition produced by the increased rainfall for the DLD period.

**Fig. 8:**
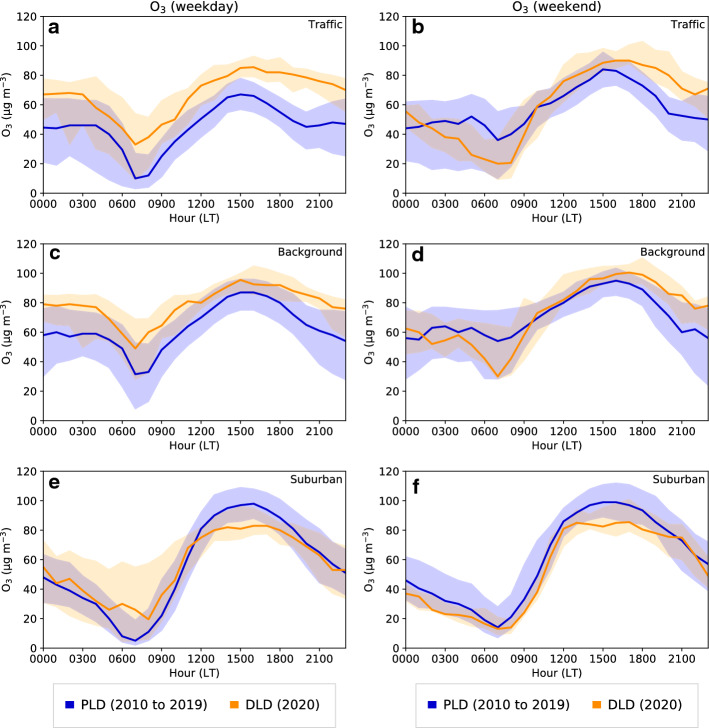
The same as in Fig. 6 but for $\hbox {O}_{{3}}$ concentrations at traffic, background, and suburban stations

Figure 8 presents the daily cycle of $\hbox {O}_{{3}}$ concentration. As already mentioned in Sect. 3.2.1, the tropospheric $\hbox {O}_{{3}}$ concentrations increased at both the traffic and background stations in the DLD period. The traffic site showed the highest increase with a sustained increase of approximately 15-20 $\upmu$g m$^{-3}$ during all weekday hours, i.e., above the 75th percentile of the PLD period. In addition, the disappearance of the $\hbox {NO}_{{2}}$ evening peak may have played a role in the slower ozone depletion in the evening and night-time. The behaviour at the urban traffic and the urban background sites was similar in the DLD period and previous differences during the PLD period were reduced, for instance, in the 0600–0900 LT concentration reductions and in the 1500 LT maximum value.

Some of the differences observed in the PLD period between weekends and weekdays (smaller $\hbox {NO}_{{2}}$ and PM10 peaks) at both stations were suppressed during the DLD period. Regarding ozone, the usual increase in weekend concentration during the daytime in urban areas still occurred, except in the early morning when the $\hbox {O}_3$ concentrations in the DLD period strongly decreased. This can be attributed to the fact that the concentration differences between weekends and weekdays were mostly suppressed during lockdown thus resulting in similar behaviours for both. Conversely, during the pre-lockdown weekends ozone was not depleted in urban areas because of the absence of the morning $\hbox {NO}_{{2}}$ peak.

#### Effects on Tropospheric Ozone

**Fig. 9:**
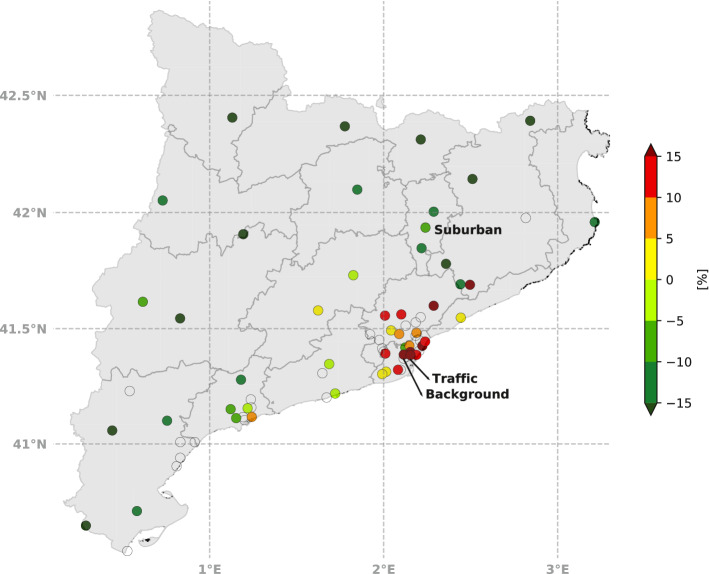
Relative difference in the mean concentration of $$\hbox {O}_{{3}}$$ during the 2010–2019 PLD period and the 2020 DLD period. Each station is represented by a coloured circle indicating the percentage of increase (orange and red colours) or decrease (green colours) in ozone. The percentage was calculated as ([$$O_{3_{DLD}}]-[O_{3_{PLD}}])/[O_{3_{PLD}}]$$ $$\times $$ 100. Transparent circles indicate that the pollutant was not measured at the station

The daily cycle of ozone concentrations at a suburban station located downwind of the Barcelona urban area was analyzed in order to assess the influence of the reduction of $\hbox {NO}_{{x}}$ and its role as an $\hbox {O}_{{3}}$ precursor. In contrast to the rise in ozone levels observed at the urban traffic and urban background monitoring sites, in 2020 there was a small decrease in ozone concentrations at the suburban site (Fig. 8e, f). On weekdays, a decline in concentration was detected between the PLD and DLD periods (58 $\upmu$ g m$^{-3}$ to 57 $\upmu$g m$^{-3}$). There was a balance between a lower ozone depletion between 0600 LT to 0900 LT due to the absence of titration during these hours as the $\hbox {NO}_x$ emissions were reduced, and a strong decrease in the daily hourly maximum, from 97 $\upmu$g m$^{-3}$ to 83 $\upmu$g m$^{-3}$, a 14% reduction (Table 5). On weekends, the PLD and DLD periods presented a similar tendency during night-time, again with a strong decrease in hourly maximum concentrations, from 100 $\upmu$g m$^{-3}$ to 87 $\upmu$g m$^{-3}$, a 13% reduction. To further illustrate the ozone behaviour, Fig. 9 shows a map including all available pollutant stations showing relative differences in tropospheric ozone concentration between the DLD and PLD means, where positive (negative) values indicate an increase (decrease) in ozone concentrations during lockdown. Two different sensitivity regimes were observed according to the type of area of the stations. Most of the urban stations showed an increase in ozone concentration in the DLD period (as shown in Figs. 5c, 8c, d), whereas rural stations tended to show a decrease in ozone concentrations. Based on the sensitivity regime explanation, in rural environments where the VOCs/$\hbox {NO}_x$ ratio was relatively high, a reduction in $\hbox {NO}_x$ led to a reduction in ozone (as shown in Fig. 8e, f). Conversely, in urban environments the reduction in $\hbox {NO}_x$ implies an increase in ozone concentrations. A decrease in VOCs during the DLD period could also have played a role, although variations in VOC emissions were not quantified in this study.

## Discussion

A statistical analysis of meteorological conditions at the UB Barcelona and AWS sites was conducted to compare the dispersion conditions between the 2020 DLD and 2016–2019 PLD periods at the local scale of Barcelona and its surroundings. Dispersion conditions in the vertical dimension, determined by the computed PBL depth, were similar in both periods, but slightly better in 2020. The surface and upper-level wind speeds were also comparable in the periods studied, but the wet deposition in March and April 2020 was greater than the 2016–2019 mean. Nevertheless, the number of rainy days per analyzed period was similar, leading to the likelihood of similar pollutant removal from the atmosphere in both periods. Although some authors have pointed out that differences in meteorological conditions between 2020 and 2019 could have played a role in the drastic reduction in $\hbox {NO}_2$ concentrations (Baldasano 2020; Tobías et al. 2020), the local-scale meteorological analysis conducted here suggests that the meteorological factors involved in vertical and horizontal dispersion conditions had a limited effect on the reduction in pollutant concentrations in Barcelona and its surroundings. These results are consistent with those of Petetin et al. (2020) who also demonstrated that the changes in $\hbox {NO}_2$ concentrations during lockdown were not directly related to the variability in meteorological conditions in Spain. Conversely, Goldberg et al. (2020) analyzed TROPOMI satellite-based $\hbox {NO}_{{2}}$ data including wind speed and direction from ERA5 reanalysis and revealed that meteorological patterns were especially favourable for low $\hbox {NO}_{{2}}$ concentrations in the United States.

The strong decrease in $\hbox {NO}_{{2}}$ concentrations can be linked to the reduction in anthropogenic activities in Barcelona. Reports from the regional Government (GENCAT 2020) indicate an abrupt reduction in the traffic emission sector, up to 70% on weekdays and 95% at weekends on main roads, 55% reduction in maritime transport operations, and between 95% to 97% decrease in air traffic operations.

Measurements of $\hbox {NO}_{{2}}$ column height taken by ESA and NASA satellites (Muhammad et al. 2020) show reductions of up to 30% in some of the epicentres of COVID-19 such as China, Italy, Spain, and the United States. Data from the European Environment Agency (EEA 2020) indicate that Rome, Paris, Toulouse, and Madrid traffic stations presented a reduction between 50% and 60% in $\hbox {NO}_{{2}}$ concentrations, in a comparison between 2018–2019 and 2020, slightly less than the percentages shown here. In the present study the calculated mean reduction in $\hbox {NO}_{{2}}$ during the lockdown period at the traffic site was higher (66%) than that reported by Baldasano (2020) and Tobías et al. (2020), probably because we considered a longer period for the PLD conditions (2010–2019). The mean percentage decrease in PM10 was similar to that found in previous studies (around 30%). In agreement with Briz-Redón et al. (2020), Tobías et al. (2020), Viteri et al. (2020) and Sulaymon et al. (2021), ozone concentration increased in urban areas during the 2020 lockdown, associated with the reduction in $\hbox {NO}_2$ levels. In contrast, ozone decreased in rural areas located downwind of the urban emission sources.

## Conclusions

Meteorological dispersion conditions including vertical and horizontal dispersion and wet deposition differed slightly between the PLD and DLD periods in Barcelona. Surface and boundary-layer wind speeds were similar in both periods, leading to a comparable horizontal-dispersion capacity of the atmosphere. Regarding vertical dispersion, the PBLH increased slightly in the DLD period (a median of 1090 m PLD and 1140 m DLD for the RAOB algorithm and 880 m PLD and 960 m DLD for the CEIL method at midday), giving a slightly greater volume of air in which pollutants can be diluted. In addition, the number of rainy days within the periods analyzed was similar (0.33 in 2016–2019, 0.34 during 2020) although rainfall amount was higher during 2020.

Our results indicate that the variation in pollutants in Barcelona was mainly caused by the emission changes that occurred during lockdown. As these emission changes were mostly caused by restrictions on mobility and by a reduction in productive activities, there was a reduction in $\hbox {NO}_{{2}}$ and PM10 levels detected at the traffic and background stations. This was particularly evident at the traffic station, with a mean $\hbox {NO}_{{2}}$ reduction from 57 $\upmu$g m$^{-3}$ in the PLD period to 19 $\upmu$g m$^{-3}$ in the DLD period (66% fractional decrease), and a PM10 reduction from 28 $\upmu$g m$^{-3}$ in the PLD period to 18 $\upmu$g m$^{-3}$ in the DLD period (36% fractional decrease). The differences between weekdays and weekends decreased to the extent that there were similar pollutant concentrations in both periods during the lockdown. Furthermore, the afternoon $\hbox {NO}_{{2}}$ peak at the traffic site was suppressed and the morning PM10 peak magnitude was lowered during lockdown.

Tropospheric ozone showed different patterns of behaviour according to the degree of urbanization close to the measurement site, leading to a certain ozone regime sensitivity driven by the limiting precursor at the station. Thus, urban areas showed an increase in ozone concentrations due to the $\hbox {NO}_{{x}}$ reduction, while rural stations ($\hbox {NO}_{{x}}$-sensitive areas) presented a decrease or a stabilization of the ozone mean value of the daily mean ozone and its daily maximum. The traffic station produced the most extreme example of the former behaviour, with the standard for the daily maximum of the 8-h rolling means of the ozone guideline being exceeded during certain periods. On the other hand, the suburban station showed a decrease in the mean of hourly maximum ozone values from 97 $\upmu$g m$^{-3}$ in the PLD period to 83 $\upmu$g m$^{-3}$ in the DLD period, producing the well known weekend effect in rural areas, where drastic reductions in $\hbox {NO}_2$ in urban areas lead to a reduction in ozone at rural sites located downwind from the urban emitting sources. Furthermore, because of the reduction in $\hbox {NO}_{{x}}$ precursors, night-time depletion also diminished, giving higher minima in the DLD period. This was noticeable on weekdays, when the difference in emissions between the PLD and DLD periods was more remarkable. It should be noted that the results presented here not only complement previous air quality COVID-19 lockdown studies but also provide insights into the effects of road traffic reduction under different meteorological conditions, which may be useful for planning or assessing future regulatory traffic-related air pollution measures.

Future work may include an analysis of different methodologies to estimate PBL depths for different meteorological conditions. In addition, broadening the meteorological variables measured would extend the scope of the analysis. A meteorological-dispersion factor analysis in other locations with similar instrumentation availability, at least a radiosonde and a ceilometer, would be valuable to confirm or refute the validity of the results collected around the urban areas affected by COVID-19.
